# Leopard (*Panthera pardus*) predation on a red-tailed monkey (*Cercopithecus ascanius*) in the Issa Valley, western Tanzania

**DOI:** 10.1007/s10329-018-0700-9

**Published:** 2018-11-17

**Authors:** Edward McLester, Kyle Sweeney, Fiona A. Stewart, Alex K. Piel

**Affiliations:** 1 School of Natural Sciences and Psychology, Liverpool John Moores University, Byrom Street, Liverpool, L33AF UK; 2 Greater Mahale Ecosystem Research and Conservation Project, Box 60118, Dar es Salaam, Tanzania

**Keywords:** Guenon, Savanna-woodland mosaic, Predator habituation, Anti-predator behavior

## Abstract

Predation is predicted to be an important selection pressure for primates. Evidence for this hypothesis is rare, however, due to the scarcity of direct observations of primate predation. We describe an observation of leopard (*Panthera pardus*) predation on a red-tailed monkey (*Cercopithecus ascanius schmidti*) at the Issa Valley, a savanna-woodland mosaic landscape in western Tanzania. We compare rates of evidence of leopard presence between Issa and other primate study sites in sub-Saharan Africa. An increase in direct observations of leopards at Issa in recent years suggests that leopards may be habituating to researcher presence.

## Introduction

Predation is predicted to be a critical selection pressure in primate evolution (Anderson 1986; Treves 1999; Zuberbühler and Jenny 2002). For example, predator avoidance is a primary explanation for the evolution of group living—an otherwise disadvantageous strategy given increased rates of intra-group feeding and mate competition (Isbell 1991; Majolo et al. 2008). Group-living primates benefit from increased collective vigilance, better defence against predators (e.g. mobbing), and greater dilution of risk among group members (Morse 1977; Boinski et al. 2000; Treves 2000). Despite this, larger groups are more conspicuous and more likely to be detected by predators (Boinski et al. 2000). While the evolutionary significance of predation is therefore of interest, testing the extent of predation as a selective pressure is difficult due to the rarity of direct observations of predation events.

Multiple factors likely influence primate vulnerability to predation. Habitat structure should determine optimal opportunities for predators as well as prey vulnerability (Isbell 1994). For example, harpy eagles (*Harpia harpyja*) may preferentially hunt primates in clearings or open canopy forest (Eason 1989), and chacma baboons (*Papio ursinus*) avoid vegetation types that are associated with higher predation risk (Cowlishaw 1997). The diversity and densities of predators should also affect encounter and predation rates (Anderson 1986). As such, for primates in open habitats (e.g. savanna-woodland mosaics) predation pressures may differ compared to closed canopy, densely vegetated habitats (e.g. tropical forests; Crook and Gartlan 1966; Anderson 1986; Dunbar 1988; Cords 1990; Isbell 1994). The diversity of potential primate predators also varies between open and closed environments, with open habitats hosting species that are no longer found or have never been historically present in tropical forests (e.g. African wild dogs, *Lycaon pictus*; spotted hyenas, *Crocuta crocuta*—Baldwin et al. 1981; Stewart and Pruetz 2013).

We describe an incident of predation on a red-tailed monkey (*Cercopithecus ascanius schmidti*), a species that typically inhabits primary and secondary forests, as well as open habitats (Sarmiento et al. 2001). Specifically, we observed a leopard (*Panthera pardus*) that preyed upon on a single monkey at the Issa Valley, a savanna-woodland mosaic landscape in western Tanzania.

## Methods

The Issa Valley study site is located approximately 90 km inland of Lake Tanganyika and equidistant between Gombe and Mahale Mountains National Parks. Research at Issa focuses on an approximately 60 km^2^ area of steep valleys and flat plateaus at an elevation of 1150–1712 m. The site has no formal protection status. The nearest settlement to the site (Mishamo) is 20 km away. Human presence in the study area occurs, primarily due to illegal poaching, logging, and cattle herding in the peripheries of the site (Piel et al. 2015). Climate is characterized by two distinct seasons: a wet season during November–April and an extended dry season (< 100 mm total monthly rainfall) during May–October (Hernandez-Aguilar 2009). Mean total annual rainfall was 1012 mm (range 760–11276 mm) in 2013–2015 (unpublished data). Vegetation is a savanna-mosaic, consisting primarily of miombo woodland dominated by *Brachystegia* and *Julbernardia* spp. with a savanna grass understory (Piel et al. 2017). Remaining vegetation is made up of a small proportion (4% cover) of evergreen and semi-deciduous gallery forest found in thin strips, as well as swamps and open grasslands. Canopy cover is mostly closed in gallery forest, and mostly open in woodland.

Red-tailed monkeys at Issa have been habituated since 2012. In 2018, two study groups (K1 and K2) were followed by two-person research teams for 5–10 days per alternate month. At the time of this study, K1 group numbered approximately 30 individuals and K2 group numbered 10 individuals. A single adult male was present in each group. To our knowledge, this is the only long-term study of red-tailed monkeys in a savanna-mosaic habitat.

Possible predators of red-tailed monkeys at Issa include five large carnivores: leopards, lions (*P. leo*), African wild dogs, East Africa black-backed jackal (*Canis mesomelas schmidti*), and spotted hyenas. Although these species are infrequently seen, African wild dogs have previously been observed to investigate a chimpanzee (McLester et al. 2016) and all five species are considered capable of preying on primates (Iwamoto et al. 1996; Stewart and Pruetz 2013). At least one species of raptor present at Issa that is known to prey upon red-tailed monkeys is the crowned hawk-eagle (*Stephanoaetus coronatus*—Skorupa 1989; Struhsaker and Leakey 1990; Mitani et al. 2001). Other likely prey for carnivores and raptors at Issa include duikers (*Philantomba* and *Sylvicapra* spp.), hyraxes (*Heterohyrax* and *Dendrohyrax* spp.), cane and giant pouched rats (*Thryonomys* and *Cricetomys* spp.), hares (*Lepus* spp.), bushbuck (*Tragelaphus scriptus*), Lichtenstein’s hartebeest (*Alcelaphus lichtensteinii*) and antelopes (*Hippotragus* spp.; Stewart 2011). Chimpanzees are also sympatric with red-tailed monkeys at Issa, and although they have never been observed to prey upon monkeys at this site, they are known to consume a range of other animals (e.g. blue duiker, *P. monticola*; bush pig, *Potamochoerus porcus*; cane rat—Ramirez-Amaya et al. 2016; unpublished data). Potential snake predators of red-tailed monkeys at Issa include African rock pythons (*Python sebae*), black mambas (*Dendroaspis polylepis*), black-necked spitting cobras (*Naja nigricollis*), and puff adders (*Bitis arietans*), as per observations of predation involving cercopithecine species from other sites (e.g. Isbell 1990; Barrett et al. 2004; Isbell 2006; Foerster 2008).

## Observation

On 10 March 2018, KS and a field assistant MM followed K1 group from 07:00. At around 18:00, the group travelled along a strip of gallery forest adjacent on either side to miombo woodland. The width of the strip varied between 40 and 120 m. Forest canopy height at this location was approximately 20–25 m over the river and 5–15 m bordering the woodland. Woodland vegetation consisted of thin trees with canopy height of approximately 10 m and vegetation on the ground was comprised of short (< 1 m) grass, moss, and bushes. Horizontal group spread (greatest distance between two individuals) was at least 50 m, with a majority of the group located in forest and a small number of individuals located in the woodland. The group was not associating with any other species. All of the monkeys in sight of the researchers were arboreal and positioned between 5 and 10 m high.

At 18:04, KS and MM were following different focal monkeys to the prey individual and were positioned approximately 50 and 30 m from the location of the interaction, respectively. MM observed a single, small- to medium-sized leopard approach the group terrestrially and slowly through the woodland, using a tree trunk for cover at one point. MM first observed the leopard when it was approximately 20 m from the group and at one point observed that the leopard appeared to look directly at her. MM was not able to identify the age or sex of the leopard. Approximately 8–10 m from the prey, the leopard continued rapidly through the woodland, climbed a vertical trunk, and seized the individual approximately 4 m above ground in a 5–10 m *Monopetalanthus richardsiae* tree, and carried the monkey back through the woodland into the forest. The researchers were not able to identify the age-sex class of the prey. The closest neighboring monkey was approximately 1 m away from the victim at the time of the attack. The prey individual was located on the relative edge of the group but was not the most peripheral individual—i.e. other members of the group were located around the individual in horizontal space. The tree in which the prey individual was attacked was located in woodland approximately 10 m away from the forest. Local canopy cover was sufficient for monkeys to move between trees without travelling terrestrially. The researchers identified scratches in the bark up to 1.7 m high that were likely to have been left by the leopard’s claws.

In response to the attack, the adult male and numerous females and juveniles climbed higher into the canopy. The male produced ka-train or “hack” alarm calls, and numerous females and juveniles produced ka-trains and chirps (Cords and Sarmiento 2013). The researchers did not observe any monkeys mobbing the leopard. All alarm vocalizations had subsided by 18:25. The group continued to travel primarily within and following the forest strip in the same direction as prior to the interaction. By 18:30, the researchers observed members of the group foraging arboreally within 5 m of the ground.

The location of the interaction was in the approximate core of K1 group’s range at the intersection of three major valleys and where the monkeys frequently travel in order to access different valleys (unpublished data). K1 group visited this location at most 18 days prior to the attack during six consecutive follow days conducted the previous month. The group next travelled through this location 2–8 days later.

## Discussion

This encounter is the first direct observation of leopard predation on a red-tailed monkey at the Issa Valley, despite over 2800 h of focal follows since January 2013. Evidence of leopard presence is observed consistently at Issa, although less frequently than at primarily forested sites (Table 1). For example, Hernandez-Aguilar (2009) and Stewart and Pruetz (2013) observed evidence once per 2 weeks and once per month, respectively, during two of the first studies to be conducted at Issa (see also Russak 2014). In recent years, direct observations of leopards have become more common. Researchers encountered leopards eight times in 2015, compared with two observations made in 2012–2014 (unpublished data). Although anecdotal, a range of leopard responses to researchers have been observed including fleeing, staying and watching researchers, and growling (EM personal observation; as per Sweanor et al. 2005). That the leopard in this observation appeared to detect one researcher and still continued the attack could suggest that leopard habituation to researcher presence is increasing at Issa.

**Table 1:** Rates of previously observed evidence of leopard presence at primate study sites in sub-Saharan Africa

Country	Study site	Site area (km^2^)	Primary habitat	Study period (length)	Number of observations	Mean rate of evidence of leopard presence	References
Botswana	Moremi Wildlife Reserve	4610	Seasonally inundated grassland and woodland	1977–1980 (30 months)	9 predation attempts (direct observations or scats near carcass)	0.3/month	Busse (1980)
Central African Republic	Dzanga-Sangha Special Reserve & Dzanga-Ndoki NP	4381	Semi-deciduous forest	1992–1994 (25 months)	160 scats	6.4/month	Ray and Sunquist (2001)
Democratic Republic of Congo	Edoro Study Area, Ituri Forest	45	Semi-evergreen forest	1988–1989 (6 months)	222 scats	37/month	Hart et al. (1996)
	Lui Kotale, Salonga NP	65	Evergreen forest	2004 (2 months)	–	0.11 tracks or scratchings/km	D’Amour et al. (2006)
Gabon	Lopé NP	4964	Semi-evergreen forest	1993–2001 (99 months)	196 scats	2/month	Henschel et al. (2005)
Ivory Coast	Taï NP	3300	Evergreen forest	1992–1994 (13 months)	200 scats	15/month	Zuberbühler and Jenny (2002)
				1980–1981 (15 months)	215 scats	14/month	Hoppe-Dominik (1984)
Kenya	Amboseli NP	392	*Acacia* spp. savanna woodland	1983; 1986–1987	–	0.63 direct observations/month	Isbell (1990)
	Segera Ranch, Laikipia	170	*Acacia* spp. savanna woodland	1998–1999 (130 days)	9 direct observations or tracks	2.1/month	Jaffe and Isbell (2010)
				1997–1999 (22 months)	6 direct observations, scats, or tracks	0.27/month	Isbell and Enstam (2002)
Tanzania	Mahale Mountains NP	1613	Semi-deciduous forest; miombo woodland	2012 (41 days)	142 scats	104/month	Nakazawa et al. (2013)
	Mt. Rungwe Nature Reserve & Kitulo NP	563	Montane forest and grassland	2003–2010	76 scats	0.03/km	De Luca and Mpunga (2018)
	Issa Valley	60	Miombo woodland; evergreen riverine forest	2008–2009 (12 months)	12 direct observations, vocalizations, scats, or tracks	1/month	Hernandez-Aguilar (2006)
				2001–2003 (21 month)	–	2 – 4 vocalizations, scats, or tracks/month	Hernandez-Aguilar (2006)
	Udzungwa Mountains NP	1999	Montane evergreen forest; deciduous woodland	2001–2002 (13 months)	28 scats	0.03/km	De Luca and Mpunga (2005)

Only sites at which leopards were extant at the time of study are included (NP: national park)

We did not observe the red-tailed monkey preyed upon to exhibit the greatest predation risk predicted by hypotheses of anti-predator behavior. For example, to maximize the chances of successful predation, predators should target the single most vulnerable animal in a group (Hamilton 1971). Frequently, this individual is isolated or on the periphery of the group (e.g. Quinn and Cresswell 2006; Josephs et al. 2016). In our observation however, the prey individual was neither isolated nor the most peripheral individual. It is possible that while the prey individual was less exposed than the most peripheral individual, it was also less vigilant and therefore more easily targeted by the leopard. Similarly, the leopard also attacked an individual from the larger of the two study groups, which does not support the hypothesis that smaller groups are more vulnerable to predation than larger groups (Janson and Goldsmith 1995; Stanford 1995). More data are needed to investigate whether open habitat-dwelling primates exhibit habitat-specific anti-predator behavior compared to primates in closed habitats.
